# Impact of nursing interventions based on a multidisciplinary team (MDT) model on recurrence rate and treatment compliance in patients with gastrointestinal bleeding

**DOI:** 10.1097/MD.0000000000049067

**Published:** 2026-06-19

**Authors:** Kexia Wang, Pengfei Sun

**Affiliations:** a Shengzhou People’s Hospital (Shengzhou Branch of the First Affiliated Hospital of Zhejiang University School of Medicine), Shengzhou City, Zhejiang Province, China; b Digestive Medicine Department, Shaoxing People’s Hospital, Shaoxing City, Zhejiang Province, China.

**Keywords:** gastrointestinal bleeding, multidisciplinary collaboration, nursing, recurrence rate, treatment compliance

## Abstract

This study aimed to investigate the impact of nursing interventions based on a multidisciplinary team (MDT) model on recurrence rate and treatment compliance in patients with gastrointestinal bleeding (GIB). A retrospective cohort study was conducted, enrolling 211 patients with acute GIB admitted from June 2022 to June 2024. Patients were divided into 2 groups according to whether they received MDT nursing care. Propensity score matching was used to control confounding bias, and a total of 96 patients (48 in the MDT group and 48 in the routine nursing group) were included in the analysis. The MDT nursing model consisted of team collaboration, joint ward rounds, staged health education, and continuity follow-up after discharge. Recurrence rate, treatment compliance, length of hospital stay, complications, and satisfaction during the 3-month follow-up period were compared between the 2 groups. After matching, baseline characteristics were balanced and comparable between the 2 groups (all *P* > .05). The MDT group had significantly lower recurrence rate (12.5% vs 33.3%, risk ratio = 0.38, 95% confidence interval: 0.16–0.85) and overall complication rate (12.5% vs 29.2%) compared with the routine care group (both *P* < .05). The MDT group also showed significantly better medication compliance (83.3% vs 58.3%, odds ratio [OR] = 3.57), dietary compliance (77.1% vs 54.2%, OR = 2.83), and follow-up compliance (85.4% vs 66.7%, OR = 2.91) (all *P* < .05). Meanwhile, the MDT group had a shorter average hospital stay (9.2 ± 3.1 vs 11.0 ± 3.8 days, *P* = .013), and higher overall satisfaction (91.7% vs 75.0%) and care experience scores (9.1 ± 0.8 vs 8.2 ± 1.1) (all *P* < .05). Subgroup analysis showed that the risk-reducing effect of MDT nursing on recurrence was more pronounced in patients aged ≥65 years and those with upper GIB (interaction *P* < .05). Nursing interventions based on the MDT model effectively reduce recurrence risk, improve treatment compliance, shorten hospital stay, decrease complications, and enhance patient satisfaction in patients with GIB. Its comprehensive benefits are significantly superior to routine nursing, with particularly greater advantages in elderly patients and those with upper gastrointestinal bleeding.

## 1. Introduction

Acute gastrointestinal bleeding (GIB) is a common gastrointestinal emergency worldwide, with a persistently high annual incidence, posing a major public health challenge.^[1]^ Its clinical manifestations range from mild melena to life-threatening hemorrhagic shock, with rapidly changing conditions. Even after successful initial hemostatic treatment, patients still face a significant risk of rebleeding. Literature has reported that the 6-week rebleeding rate of acute non-variceal upper GIB is as high as 10–20%, while the rebleeding risk of esophagogastric variceal rupture bleeding is even higher, greatly affecting patients’ long-term prognosis and quality of life.^[2,3]^

The reasons behind this high recurrence rate are multifactorial. In addition to the nature of the lesion itself (such as Forrest classification and variceal severity) and the patient’s baseline conditions (such as liver and kidney function and coagulation function), post-discharge treatment compliance has been widely recognized as an independent risk factor for predicting rebleeding, yet it remains the most easily overlooked link in clinical management.^[4]^ The treatment of GIB is a continuous process, involving long-term regular use of proton pump inhibitors, nonselective β-blockers for portal hypertension, as well as strict adherence to dietary instructions such as liquid/soft diets and avoidance of coarse or irritating foods. However, during the transition from the highly monitored hospital environment to home-based self-management, patients often experience medication interruption, poor dietary compliance, and loss to follow-up due to insufficient knowledge, memory decline, confusion caused by polypharmacy, financial burden, or lack of effective supervision. These issues greatly weaken the effectiveness of initial treatment and lay hidden risks for rebleeding.^[5]^

Traditional nursing models are often “fragmented,” with attending physicians and nurses working within their respective scopes of responsibility, lacking systematic integration. This model is insufficient in addressing patients’ complex and diverse health needs, especially in areas such as precise medication guidance, individualized nutritional support, and continuity of follow-up across the “hospital–community–home” continuum.^[6]^ The multidisciplinary team (MDT) model is an innovative strategy to address this challenge. It breaks disciplinary boundaries by forming a core team that includes gastroenterologists, specialist nurses, clinical pharmacists, dietitians, and even endoscopists, who jointly assess, decide, and implement integrated diagnosis, treatment, and nursing plans, ensuring that patients receive the most professional support at every stage from the acute rescue phase to the recovery and rehabilitation phase.^[7,8]^ Existing evidence indicates that the MDT model can effectively reduce complications and improve patient outcomes in disease areas such as stroke and cancer.^[9]^

However, despite the wide recognition of the MDT concept, strong evidence from rigorously designed studies regarding its effectiveness in the specific emergency context of acute GIB – particularly its comprehensive impact on recurrence rate and multidimensional treatment compliance (medication, diet, follow-up) – remains lacking. Most related reports still remain at the level of experiential summaries or are limited by small sample sizes, inadequate control groups, or failure to effectively control confounding bias.^[10]^

Therefore, to fill this evidence gap, this study aims to use a retrospective cohort study design and apply propensity score matching (PSM), an advanced statistical method that simulates the environment of randomized controlled trials and maximizes the control of selection bias, to scientifically and accurately evaluate the real-world effectiveness of MDT-based nursing interventions in reducing recurrence rate and improving treatment compliance in patients with acute GIB. The results of this study will provide essential data support and decision-making evidence for the standardized clinical promotion of this nursing model.

## 2. Methods

### 2.1. Study design

This single-center retrospective cohort study was approved by the Ethics Committee of Shaoxing People’s Hospital. This study was a single-center retrospective cohort study conducted at Shaoxing People’s Hospital. This study adopted a retrospective cohort design, collecting a total of 211 patients with acute GIB who were diagnosed and hospitalized in our hospital from June 2022 to June 2024. Patients were divided into an MDT nursing group and a routine nursing group based on whether they had previously received nursing interventions under the MDT model.

Considering potential differences in baseline characteristics between the 2 groups, PSM was applied to reduce selection bias and the influence of confounding factors. Matching variables included age, sex, bleeding site, underlying diseases, history of gastrointestinal disorders, and other common confounders. A 1:1 matching ratio was used, and the nearest-neighbor matching method (caliper = 0.2) was employed. A total of 96 successfully matched patients were obtained, including 48 in the MDT group and 48 in the control group.

As this was a single-center retrospective cohort study, the sample size was not determined a priori but depended on the number of eligible patients admitted during the study period. All consecutive patients meeting the inclusion and exclusion criteria between June 2022 and June 2024 were screened for eligibility. After PSM, 96 patients (48 in each group) were included in the final analysis. To evaluate whether the matched sample provided reasonable statistical informativeness for the primary outcome, a post hoc power consideration was performed based on the observed recurrence rates in the 2 groups (12.5% in the MDT group vs 33.3% in the routine nursing group). With a 2-sided α of 0.05, this matched sample size provided acceptable power to detect a between-group difference of this magnitude in the primary outcome. Nevertheless, the sample size remained limited for subgroup analyses and some secondary endpoints, and these results should therefore be interpreted as exploratory.

### 2.2. Inclusion and exclusion criteria

#### 2.2.1. Inclusion criteria

First hospitalization in our hospital for acute GIB; age between 50 and 80 years; diagnosis of acute GIB confirmed by clinical presentation, laboratory tests, and endoscopy (gastroscopy or colonoscopy), including upper and lower GIB; complete clinical medical records, with at least 3 months of follow-up information after discharge (including recurrence, medication compliance, and readmission records).

#### 2.2.2. Exclusion criteria

Secondary GIB caused by trauma, surgery, or other iatrogenic factors; patients with advanced malignant tumors, severe hepatic or renal failure (such as Child-Pugh class C or end-stage renal disease), severe coagulation dysfunction, or other serious comorbidities that significantly affect prognosis and survival; patients with severe cognitive impairment, psychiatric disorders, or communication barriers that prevent the assessment of treatment compliance; patients who died or were discharged automatically within 24 hours after admission and thus could not complete the basic treatment process; patients with missing clinical data or loss to follow-up that precluded effective data analysis.

### 2.3. Nursing methods

Retrospective analysis showed that all enrolled patients were initially admitted to the intensive care unit (ICU) due to critical illness, where they received close monitoring of vital signs and supportive treatment. After their vital signs stabilized and bleeding was preliminarily controlled (generally within one week), they were transferred to the general ward of the Department of Gastroenterology for continued treatment. According to medical record documentation, the nursing models received by patients during hospitalization differed; therefore, they were divided into 2 groups accordingly.

#### 2.3.1. *Control group (routine nursing group*)

Medical record data showed that patients in this group received routine nursing care in the Department of Gastroenterology. The nursing practices mainly followed standard medical orders, including:

##### 2.3.1.1. Basic nursing and condition monitoring

Records showed that the nursing staff instructed patients to maintain strict bed rest and assisted them in adopting appropriate positions; promptly established venous access, executed medical orders for blood transfusion, fluid replacement, and hemostatic drugs (such as somatostatin, with documentation indicating the use of an infusion pump to ensure steady infusion), and observed and recorded adverse drug reactions; meanwhile, closely monitored patients’ vital signs, mental status, signs of bleeding, and accurately recorded 24-hour intake and output.

##### 2.3.1.2. Dietary and daily-life guidance

Nursing documentation indicated strict fasting during active bleeding; after bleeding cessation, staff guided patients to gradually transition to a high-calorie, high-vitamin liquid diet according to medical orders. Nursing staff also provided guidance on physical activity, emphasizing slow movements to prevent falls, and assisted with basic care such as eating, personal hygiene, and elimination.

##### 2.3.1.3. Routine health education

According to the health education records, responsible nurses provided education to patients and their families regarding disease knowledge, lifestyle modification, dietary control, correct medication use, and self-monitoring. Medical records also included notations instructing patients to “notify the physician promptly if abnormalities occur.”

#### 2.3.2. *Observation group (multidisciplinary/MDT nursing group*)

Medical record analysis indicated that, on the basis of routine nursing, patients in this group received systematic nursing interventions based on the MDT model. By reviewing departmental work records, handover reports, and follow-up archives, the intervention content could be summarized as follows.

##### 2.3.2.1. MDT team composition and implementation

Departmental documents indicated the establishment of an MDT collaborative group composed of the head nurse, specialist physicians, clinical pharmacists, dietitians, endoscopy nurses, and several responsible nurses. Training records showed that team members received training on relevant workflows before the implementation of the intervention.

##### 2.3.2.2. MDT joint ward rounds and assessment

Comparison of nursing ward-round records showed that this group received more frequent joint ward rounds (typically once every other day), jointly conducted by attending physicians and responsible nurses, with more comprehensive assessment content that included soliciting patient opinions. Medication review records by clinical pharmacists and minutes of the team’s weekly meetings further confirmed their role in medication guidance and risk screening.

##### 2.3.2.3. Stage-based MDT health guidance

##### 2.3.2.3.1. Admission transition phase

Handover records showed explicit documentation of participation by endoscopy nurses within 24 hours after patients were transferred to the general ward, including the transmission of post-endoscopy specific medical orders and key nursing points.

##### 2.3.2.3.2. Discharge preparation phase

Discharge assessment records and health-education documents confirmed that approximately 24 hours before discharge, clinical pharmacists and dietitians jointly conducted focused discharge guidance and developed written individualized medication and dietary plans.

##### 2.3.2.3.3. Post-discharge MDT continuous follow-up

Retrieved follow-up records indicated that dedicated nurses conducted follow-up for at least 3 months through telephone calls and WeChat groups. Chat records in WeChat groups confirmed the dissemination of health-education materials and interactive Q&A processes; for complex issues, messages were forwarded to specialists in the corresponding departments for expert responses. For patients not using WeChat, the follow-up log contained documentation of regular telephone follow-ups.

## 2.4. Data collection

Data were collected through retrospective analysis of the electronic medical record system, nursing records, and the hospital follow-up database. To ensure data quality, 2 trained researchers independently extracted and entered the data, and inconsistencies were checked and adjudicated. The collected data included the following items:

### 2.4.1. Baseline data

Baseline data were extracted from the patients’ admission medical record system and mainly included the following information:

For demographic characteristics, we recorded age and sex of all patients.

For disease-related features, based on findings from endoscopic examinations (gastroscopy or colonoscopy), the bleeding site was classified as upper GIB or lower GIB; meanwhile, the etiology of bleeding was categorized as peptic ulcer, esophagogastric variceal bleeding, acute gastric mucosal lesions, or others according to endoscopic and clinical diagnoses.

To assess the severity of illness, we extracted the lowest hemoglobin level at admission (unit: g/L), and calculated the shock index (heart rate/systolic blood pressure) using the initial vital signs. A value >0.7 was adopted as the threshold for hemodynamic instability and processed as a binary variable.

Regarding comorbidities, combining admission diagnoses and past medical history, we recorded whether patients had hypertension, diabetes, liver cirrhosis, or coronary heart disease.

In addition, based on the nursing admission assessment records, we identified patients’ health-risk behaviors, including current smoking status and long-term alcohol consumption (defined as a daily ethanol intake exceeding 40 g for more than 5 years).

#### 2.4.2. Outcome measures

##### 2.4.2.1. Primary outcome: recurrence of GIB

The primary outcome of this study was the recurrence of GIB. Recurrence was defined as the reappearance of clinical symptoms suggestive of active bleeding – such as hematemesis, melena, or hematochezia – within the 3-month follow-up period after successful discharge, and confirmed by clinicians through comprehensive evaluation or endoscopic examination. To accurately identify recurrence events, all data were cross-validated and uniformly compiled by reviewing all rehospitalization records, emergency visit records, and scheduled outpatient follow-up records within 3 months after discharge. The use of different time windows for outcome assessment was based on clinical relevance. The primary outcome (recurrence) and treatment compliance were evaluated over a 3-month follow-up period to reflect mid-term disease control and post-discharge management effectiveness. In contrast, hospitalization-related complications were assessed during the hospital stay and within 30 days after discharge, as these events are more closely associated with acute treatment processes and early recovery. This design allows for a comprehensive evaluation of both short-term safety and mid-term effectiveness of the MDT intervention.

##### 2.4.2.2. Secondary outcome 1: treatment compliance

Treatment compliance was evaluated during the 3-month follow-up period after discharge, based on systematic follow-up records. It included the following 3 core dimensions:

##### 2.4.2.3. Medication compliance

The assessment combined subjective statements and objective verification. Patients were classified as “compliant” only if both criteria were met:

During follow-up, the patient or family clearly reported no self-discontinuation, dose reduction, or alteration in medication frequency.

Follow-up records by clinical pharmacists or responsible nurses contained no documented reports of medication misuse, missed doses, or dosing errors.

##### 2.4.2.4. Dietary compliance

This indicator was assessed by dietitians or trained responsible nurses during follow-up according to preset criteria. Judgment was based on whether the patient’s actual dietary pattern basically matched the individualized dietary plan formulated during hospitalization by the MDT team (e.g., low-fiber, low-residue, avoidance of spicy or irritating foods), with the explicit evaluation recorded in the follow-up documentation serving as the standard.

##### 2.4.2.5. Follow-up compliance

This indicator was based on objective records. Patients were considered compliant if they returned for outpatient review within 1 week before or after the first follow-up time point specified in the discharge instructions.

##### 2.4.2.6. Secondary outcome 2: hospitalization indicators and complications

Data related to hospitalization resource utilization and safety outcomes were extracted from the hospitalization summary and detailed nursing records. Length of hospital stay was calculated in days, defined as the total duration from admission date to official discharge date. Complications were monitored throughout the entire hospitalization period and within 30 days after discharge, focusing on adverse events associated with the disease itself or with diagnostic/treatment measures, including: clinically documented drug adverse reactions, malnutrition confirmed by dietitians using standardized tools (e.g., patient-generated subjective global assessment), falls or bed-accident events, and aspiration pneumonia or other clearly defined aspiration events.

In addition, the ICU readmission rate was recorded, defined as cases in which patients, after transfer from ICU to the general ward, required readmission to ICU due to worsening of the primary condition or development of new severe complications.

##### 2.4.2.7. Secondary outcome 3: patient satisfaction and care experience

Patient satisfaction and care experience data were obtained from the hospital’s standardized discharge patient satisfaction survey system, which was completed under guidance before discharge. The satisfaction survey included 4 specific domains – health education, medication guidance, dietary guidance, and post-discharge follow-up services – as well as an overall satisfaction evaluation. All satisfaction items used binary choices, with patients selecting either “satisfied” or “unsatisfied.” The criterion for overall satisfaction was that the patient explicitly selected “satisfied” in the “overall satisfaction” item.

The overall care-experience score was measured using a numerical rating scale, asking patients to provide an overall score for their care experience during hospitalization, ranging from 0 (representing “very dissatisfied”) to 10 (representing “very satisfied”).

#### 2.4.3. Subgroup analysis variables

Variables used for subgroup analyses were extracted from baseline data, including: age (categorized at 65 years), bleeding site (upper vs lower GIB), and liver cirrhosis status (yes vs no).

### 2.5. Statistical analysis

SPSS 26.0 software was used for statistical analysis. All tests were 2-sided, and a *P* value < .05 was considered statistically significant. The MatchIt package in R software (version 4.2.1) was used for PSM, applying 1:1 nearest-neighbor matching (caliper = 0.2), with matching variables including age, sex, bleeding site, and other baseline characteristics, yielding 96 matched samples for analysis. After matching, the following statistical methods were used: continuous variables conforming to a normal distribution were described as mean ± standard deviation and compared between groups using independent-samples *t* tests; categorical variables were described as frequencies and percentages and compared using the χ^2^ test or Fisher exact test. For the primary outcome (recurrence rate), the risk ratio (RR) and its 95% confidence interval (CI) were calculated, while for treatment compliance, the odds ratio (OR) and its 95% CI were calculated. Subgroup analyses used interaction tests to evaluate differences in the effect of MDT nursing across subgroups.

## 3. Results

### 3.1. Baseline characteristics of patients

A total of 211 patients were initially enrolled in this study. After PSM, 96 patients were included in the final analysis, with 48 patients in the MDT nursing group and 48 in the routine nursing group. As shown in Table 1, after matching, there were no statistically significant differences between the 2 groups in age, sex, bleeding site, admission hemoglobin level, shock index, bleeding etiology, major comorbidities, or unhealthy lifestyle behaviors (all *P* > .05), indicating good comparability between the groups.

**Table 1:** Comparison of baseline characteristics between groups after propensity score matching (n = 96).

Baseline characteristic	MDT care group (n = 48)	Conventional care group (n = 48)	Statistical value	*P* value
Demographics				
Age (yr), mean ± SD	68.1 ± 7.2	66.5 ± 8.0	*t* = 1.04	.303
Male, n (%)	31 (64.6)	28 (58.3)	χ^2^ = 0.42	.517
Bleeding-related characteristics				
Bleeding site, n (%)			χ^2^ = 0.34	.558
Upper GI bleeding	40 (83.3)	42 (87.5)		
Lower GI bleeding	8 (16.7)	6 (12.5)		
Hemoglobin on admission (g/L), mean ± SD	78.5 ± 16.8	81.2 ± 17.5	*t* = -0.77	.441
Shock index > 0.7, n (%)	18 (37.5)	15 (31.3)	χ^2^ = 0.44	.508
Etiology of bleeding, n (%)				
Peptic ulcer	18 (37.5)	22 (45.8)	χ^2^ = 0.69	.407
Esophagogastric varices	13 (27.1)	10 (20.8)	χ^2^ = 0.52	.471
Acute gastric mucosal lesion	10 (20.8)	8 (16.7)	χ^2^ = 0.28	.599
Other	7 (14.6)	8 (16.7)	χ^2^ = 0.08	.773
Major comorbidities, n (%)				
Hypertension	26 (54.2)	23 (47.9)	χ^2^ = 0.38	.536
Diabetes mellitus	16 (33.3)	13 (27.1)	χ^2^ = 0.47	.494
Liver cirrhosis	15 (31.3)	12 (25.0)	χ^2^ = 0.48	.49
Coronary heart disease	11 (22.9)	9 (18.8)	χ^2^ = 0.25	.619
Health risk behaviors, n (%)				
Current smoker	17 (35.4)	14 (29.2)	χ^2^ = 0.44	.508
Long-term alcohol use	13 (27.1)	10 (20.8)	χ^2^ = 0.52	.471

MDT = multidisciplinary team, SD = standard deviation.

### 3.2. Recurrence rate of GIB

During the 3-month follow-up period, the recurrence of GIB in the 2 groups is shown in Table 2. The recurrence rate in the MDT nursing group was 12.5% (6/48), which was significantly lower than 33.3% (16/48) in the routine nursing group, with a statistically significant difference (χ^2^ = 6.32, *P* = .012). Risk ratio (RR) analysis indicated that, compared with routine nursing care, patients receiving MDT nursing had a 62% reduction in the risk of recurrent GIB (RR = 0.38, 95% CI: 0.16–0.85).

**Table 2:** Comparison of recurrent bleeding rates between the 2 groups.

Group	Total (n)	Recurrence, n (%)	χ^2^ value	*P* value	RR (95% CI)
MDT care group	48	6 (12.5%)	6.32	.012	0.38 (0.16–0.85)
Conventional care group	48	16 (33.3%)			1.00 (reference)

CI = confidence interval, MDT = multidisciplinary team, RR = risk ratio, SD = standard deviation.

### 3.3. Treatment compliance

In terms of treatment compliance, the MDT nursing group demonstrated overall superiority (Table 3). Specifically, the MDT group showed significantly higher medication compliance (83.3% vs 58.3%; χ^2^ = 7.11, *P* = .008), dietary compliance (77.1% vs 54.2%; χ^2^ = 5.69, *P* = .017), and follow-up compliance (85.4% vs 66.7%; χ^2^ = 4.69, *P* = .030) compared with the routine nursing group. The OR indicated that the likelihood of achieving good compliance in the MDT group was 2.83–3.57 times that in the control group.

**Table 3:** Comparison of treatment compliance between the 2 groups.

Compliance category	MDT care group (n = 48)	Conventional care group (n = 48)	χ^2^ value	*P* value	OR (95% CI)
Medication compliance	40 (83.3%)	28 (58.3%)	7.11	.008	3.57 (1.45–8.81)
Dietary compliance	37 (77.1%)	26 (54.2%)	5.69	.017	2.83 (1.22–6.62)
Follow-up visit compliance	41 (85.4%)	32 (66.7%)	4.69	.03	2.91 (1.14–7.58)

CI = confidence interval, MDT = multidisciplinary team, OR = odds ratio.

### 3.4. Hospitalization indicators and complications

MDT nursing also showed significant effectiveness in improving hospitalization indicators and reducing complications (Table 4). The mean length of hospital stay in the MDT nursing group was significantly shorter than that in the routine nursing group (9.2 ± 3.1 vs 11.0 ± 3.8 days; *t* = –2.54, *P* = .013). In addition, the overall complication rate was significantly lower in the MDT group (12.5% vs 29.2%; χ^2^ = 4.04, *P* = .044), and a decreasing trend was observed in ICU readmission rate (2.1% vs 10.4%; χ^2^ = 2.84, *P* = .092).

**Table 4:** Comparison of length of hospital stay and complication rates between the 2 groups.

Indicator	MDT care group (n = 48)	Conventional care group (n = 48)	Statistical value	*P* value
Length of hospital stay (d), mean ± SD	9.2 ± 3.1	11.0 ± 3.8	t = −2.54	.013
Total complications, n (%)	6 (12.5%)	14 (29.2%)	χ^2^ = 4.04	.044
Drug-related adverse reactions, n (%)	2 (4.2%)	5 (10.4%)	χ^2^ = 1.39	.239
Malnutrition, n (%)	2 (4.2%)	4 (8.3%)	χ^2^ = 0.71	.399
Falls/bed falls, n (%)	1 (2.1%)	3 (6.3%)	χ^2^ = 1.04	.308
Aspiration pneumonia/aspiration events, n (%)	1 (2.1%)	2 (4.2%)	χ^2^ = 0.34	.559
ICU readmission rate, n (%)	1 (2.1%)	5 (10.4%)	χ^2^ = 2.84	.092

ICU = intensive care unit, MDT = multidisciplinary team.

### 3.5. Patient satisfaction and care experience

Regarding patient-reported outcomes, the MDT nursing group demonstrated significantly higher satisfaction across all dimensions compared with the routine nursing group (Table 5). The overall satisfaction rate in the MDT group was 91.7%, which was significantly higher than 75.0% in the control group (χ^2^ = 4.80, *P* = .028). Satisfaction with specific aspects – including health education, medication guidance, dietary guidance, and post-discharge follow-up services – was also significantly higher in the MDT group (all *P* < .01). The overall care-experience score was likewise significantly higher in the MDT group compared with the control group (9.1 ± 0.8 vs 8.2 ± 1.1; *t* = 4.58, *P* < .001).

**Table 5:** Comparison of patient satisfaction and care experience indicators between the 2 groups.

Indicator	MDT care group (n = 48)	Conventional care group (n = 48)	Statistical value	*P* value
Overall satisfaction, n (%)	44 (91.7%)	36 (75.0%)	χ^2^ = 4.80	.028
Satisfaction with health education, n (%)	45 (93.8%)	34 (70.8%)	χ^2^ = 8.65	.003
Satisfaction with medication guidance, n (%)	46 (95.8%)	35 (72.9%)	χ^2^ = 9.56	.002
Satisfaction with dietary guidance, n (%)	44 (91.7%)	33 (68.8%)	χ^2^ = 7.94	.005
Satisfaction with post-discharge follow-up services, n (%)	45 (93.8%)	32 (66.7%)	χ^2^ = 11.09	<.001
Overall care experience score (0–10), mean ± SD	9.1 ± 0.8	8.2 ± 1.1	t = 4.58	<.001

SD = standard deviation.

### 3.6. Subgroup analysis

Subgroup analysis using recurrence rate as the primary outcome (Table 6) showed that MDT nursing demonstrated a trend toward reducing recurrence risk across subgroups stratified by age, bleeding site, and presence or absence of liver cirrhosis. Interaction tests indicated differences in the effect of MDT nursing among subgroups. The benefit was more pronounced in patients aged ≥65 years (*P* for interaction = 0.039) and in those with upper GIB (*P* for interaction = .026). No significant difference in effect size was observed across subgroups defined by liver cirrhosis status (*P* for interaction = .445).

**Table 6:** Subgroup analysis for the primary outcome (recurrent bleeding).

Subgroup	MDT group events/total	Control group events/total	RR (95% CI)	*P* for interaction
Age				0.039
< 65 yr	2/17	5/20	0.47 (0.10–2.10)	
≥ 65 yr	4/31	11/28	0.33 (0.12–0.92)	
Bleeding site				0.026
Upper GI	4/40	14/41	0.29 (0.11–0.83)	
Lower GI	2/8	2/7	0.88 (0.16–4.58)	
Liver cirrhosis				0.445
Yes	3/15	5/12	0.48 (0.14–1.62)	
No	3/33	11/36	0.30 (0.10–0.96)	

CI = confidence interval, GI = gastrointestinal, MDT = multidisciplinary team.

## 4. Discussion

Previous studies have suggested that the MDT model can integrate the assessment and decision-making strengths of different specialties in the management of complex gastrointestinal diseases, thereby improving diagnostic and therapeutic processes, enhancing patient compliance, and reducing adverse events.^[9,10]^ However, systematic research on MDT nursing in the field of acute GIB remains relatively limited, and high-quality clinical evidence with rigorous methodological control of confounding factors is particularly lacking. In this study, through a retrospective cohort analysis and the application of PSM to control baseline confounders, we systematically evaluated the comprehensive impact of MDT-based nursing interventions on clinical outcomes in patients with GIB. The use of different outcome time windows in this study reflects the clinical distinction between short-term safety events (e.g., complications) and mid-term outcomes (e.g., recurrence and compliance), thereby enabling a more comprehensive evaluation of MDT effectiveness across different stages of patient management.

The results showed that, compared with patients receiving routine nursing care, the MDT nursing group exhibited a 62% reduction in the risk of GIB recurrence within the 3-month follow-up period. This key finding directly addresses a major clinical challenge – the high recurrence rate.^[11]^ We believe that this significant benefit may stem from the MDT model’s systematic and structured intervention pathway, which effectively disrupts the chain of risk leading to rebleeding across multiple critical points.^[12]^ Specifically, the in-depth participation of clinical pharmacists ensures the scientific and individualized optimization of medication regimens, and their continuous review and guidance reduce inappropriate medication behaviors at the source; dietitian-led nutritional management provides precise nutritional support and prevents mucosal injury triggered by improper diet; and comprehensive health education throughout hospitalization and discharge, together with continuous follow-up by dedicated nurses, significantly strengthens patients’ disease understanding and self-management capacity.^[13]^ These multidimensional collaborative interventions form a solid foundation for reducing recurrence risk.

Another important finding of this study is that MDT nursing demonstrated remarkable advantages in comprehensively improving treatment compliance. Patients showed better performance not only in medication compliance but also in dietary management and timely follow-up.^[14]^ This fully reflects the core value of the MDT model in integrating professional strengths and implementing continuous health management. Routine nursing often leads to fragmented health information due to insufficient collaboration among different professional groups,^[15]^ whereas the MDT model establishes standardized collaborative workflows, ensuring continuity and consistency in health education and enabling patients to receive comprehensive and seamless care support.^[16]^ This patient-centered service model improves both objective clinical indicators and patients’ subjective experiences – MDT patients had significantly higher overall satisfaction and dimension-specific satisfaction scores compared with the control group,^[17]^ indicating that comprehensive and individualized nursing services better meet patients’ health needs and enhance the warmth and quality of healthcare services.

From the perspective of health economics, the shortened length of hospital stay and reduced complication rate observed in the MDT nursing group suggest the potential value of this model in optimizing medical resource allocation and reducing the burden on the healthcare system.^[18]^ Although the difference in ICU readmission rates did not reach statistical significance, the downward trend observed is still noteworthy and may require studies with larger sample sizes for further validation. The subgroup analysis provides more targeted clinical insights. The particularly notable effectiveness of MDT nursing in elderly patients and those with upper GIB^[19]^ may be related to the higher recurrence risk, more complex medication regimens, and greater need for nutritional support in these groups.^[20,21]^ The MDT model is well suited to address these complex needs through more precise and intensified management, thereby achieving a greater absolute risk reduction.

However, caution is needed when interpreting the results of this study. First, although PSM was used to balance measured baseline characteristics between groups, residual confounding from unmeasured variables cannot be fully excluded. For example, factors such as patients’ socioeconomic status, health literacy, family support, or intrinsic motivation for treatment adherence were not captured in the dataset. These factors may have been unevenly distributed between groups and could potentially lead to an overestimation of the beneficial effects of MDT nursing, particularly for outcomes related to treatment compliance and follow-up adherence. Conversely, given that patients receiving MDT care often present with more complex clinical needs in real-world settings, the observed effect size might also be underestimated. Therefore, the true magnitude of the MDT effect may lie within a range influenced by both positive and negative residual biases, and the findings should be interpreted with appropriate caution. Second, this study was conducted at a single center with a relatively limited sample size, and the number of outcome events in subgroup analyses was small. In addition, multiple subgroup and secondary outcome analyses were performed without formal adjustment for multiple comparisons, which increases the risk of type I error. Therefore, these findings – particularly subgroup effects – should be considered exploratory and hypothesis-generating rather than confirmatory. Although consistent trends were observed across several outcomes, the results should not be overgeneralized. In addition, the assessment of treatment compliance partially relied on patient self-report, which may be subject to recall bias and social desirability bias. Future research may incorporate multicenter, prospective randomized controlled trial designs and use more objective compliance measurement methods (such as electronic medication monitoring devices) to further validate the conclusions of this study.

In conclusion, this study demonstrates that nursing interventions based on the MDT model can effectively reduce recurrence risk, improve hospitalization indicators, and enhance patient satisfaction by improving treatment compliance and optimizing disease management processes. This model provides a practical solution for achieving comprehensive and refined management of GIB and improving long-term outcomes, particularly among high-risk populations such as elderly patients and those with upper GIB. It is therefore worthy of further promotion in clinical practice.

## Author contributions

**Conceptualization:** Kexia Wang, Pengfei Sun.

**Data curation:** Kexia Wang, Pengfei Sun.

**Formal analysis:** Kexia Wang, Pengfei Sun.

**Funding acquisition:** Pengfei Sun.

**Writing – original draft:** Pengfei Sun.

**Writing – review & editing:** Pengfei Sun.
